# An Open Question: Is Non-Ionizing Radiation a Tool for Controlling Apoptosis-Induced Proliferation?

**DOI:** 10.3390/ijms222011159

**Published:** 2021-10-16

**Authors:** Samantha J. Hack, Luke J. Kinsey, Wendy S. Beane

**Affiliations:** Department of Biological Sciences, Western Michigan University, Kalamazoo, MI 49008, USA; samantha.j.hack@wmich.edu (S.J.H.); luke.j.kinsey@wmich.edu (L.J.K.)

**Keywords:** apoptosis, non-ionizing radiation, proliferation, reactive oxygen species (ROS), weak magnetic fields, quantum biology

## Abstract

Non-ionizing radiation is commonly used in the clinical setting, despite its known ability to trigger oxidative stress and apoptosis, which can lead to damage and cell death. Although induction of cell death is typically considered harmful, apoptosis can also be beneficial in the right context. For example, cell death can serve as the signal for new tissue growth, such as in apoptosis-induced proliferation. Recent data has shown that exposure to non-ionizing radiation (such as weak static magnetic fields, weak radiofrequency magnetic fields, and weak electromagnetic fields) is able to modulate proliferation, both in cell culture and in living organisms (for example during tissue regeneration). This occurs via in vivo changes in the levels of reactive oxygen species (ROS), which are canonical activators of apoptosis. This review will describe the literature that highlights the tantalizing possibility that non-ionizing radiation could be used to manipulate apoptosis-induced proliferation to either promote growth (for regenerative medicine) or inhibit it (for cancer therapies). However, as uncontrolled growth can lead to tumorigenesis, much more research into this exciting and developing area is needed in order to realize its promise.

## 1. Introduction

The last century has brought about revolutionary discoveries that have furthered our fundamental understanding of natural phenomena, such as ionizing and non-ionizing radiation. These discoveries have fueled the rapid development of breakthrough technologies that shape the way we live in, interact with, and understand the world around us. There is a growing amount of evidence that suggests that non-ionizing radiation (NIR) interacts with biological systems in a meaningful way. Importantly, understanding the mechanisms involved in these interactions will enable the development of powerful next-generation experimental tools and therapeutics.

Although non-ionizing radiation (such as static magnetic fields, radiofrequency magnetic fields, and electromagnetic fields) has been used diagnostically in healthcare for decades, it has been largely researched in the context of negative health outcomes [1,2]. Much of this concern centered around research on the development and propagation of cancers [3]. However, more recent reports have highlighted the potential use of NIR as a non-invasive strategy to modulate cellular activities such as tissue growth in therapeutic settings. Specifically, various forms of NIR have been shown to alter cell proliferation, differentiation, and apoptosis (programmed cell death) in experiments both outside (in vitro) and within (in vivo) living organisms [4]. For example, a combination of low-level alternating and static magnetic fields has been shown to suppress tumor development in mouse models of cancer [5]. Evidence such as this suggests that NIR may be a potential therapeutic tool for cancer and other related diseases.

This recent progress is exciting, yet our understanding of the mechanisms by which NIR induces these effects is limited in many capacities. This poses significant barriers for the implementation of new NIR therapies in clinical settings where the fine control of cell proliferation and tissue growth are key to successful treatment. In this review, we will (1) describe the role of NIR in controlling cellular activities; (2) highlight the means by which apoptosis can promote proliferation and tissue growth; (3) outline the current evidence suggesting that NIR may be a tool to control tissue growth by drawing attention to reactive oxygen species (ROS) and apoptosis-induced proliferation. Due to the cross-disciplinary nature of this subject, we have included a list of definitions for some of the terminology involved (Table 1).

## 2. Non-Ionizing Radiation and Control of Cellular Processes

Studies investigating the biological implications of exposure to different forms of NIR can be difficult to sort through given the range of phenomena that are grouped together as non-ionizing, such as radio waves, microwaves, infrared radiation, visible light, and a portion of ultraviolet (UV) radiation. Here, we will focus specifically on the beneficial effects of NIR in regulating cell fate, proliferation, differentiation, and apoptosis (reviewed in more detail in Section 4). In order to understand these data, it is first important to describe our current understanding of the mechanisms by which NIR is able to modulate cellular activities.

One of the main mechanisms through which NIR is able to produce biological effects is by influencing the generation of reactive oxygen species (ROS) [1]. Biological ROS include the superoxide anion (O_2_^−^), the hydroxyl radical (^-^OH), and hydrogen peroxide (H_2_O_2_), all of which are generated both during normal metabolism and in times of cellular stress [6]. Except for H_2_O_2_, these are all free radicals, in other words molecules with unpaired electrons. All ROS are biologically relevant, and varying ROS levels can have immense cellular consequences. High levels of ROS, which for intracellular H_2_O_2_ is greater than 100 nM, result in cell damage and disease [7]. Traditionally, this damage was observed as oxidative stress affecting DNA, protein, and lipid structures, eventually leading to carefully programmed cell death (apoptosis) [8]. These negative effects can influence disease states, as ROS have been associated with the development and progression of various cancers when apoptosis does not occur [9]. However, cellular ROS accumulation does not always result in cell death. Intermediate levels of ROS, within physiological acceptable ranges (less than 100 nM for intracellular H_2_O_2_), can act as second messengers [7]. In this way, they contribute to redox signaling (signal transduction by electron transfer). One example of this type of oxidation-reduction reaction is H_2_O_2_-mediated oxidation of cysteine residues within proteins [10]. These signals are known to positively regulate cell proliferation, differentiation, and growth [7]. Therefore, discovering innovative technologies to elicit fine control over ROS levels would be highly beneficial.

Theoretical modeling suggests that one way in which NIR can control ROS levels is by influencing the spin state of electrons, thus altering levels of free radicals (see Section 4 for a detailed explanation) [11]. These increases or decreases in ROS generation can result in changes in redox signaling, directly affecting cellular activities. Thus, NIR has been suggested as a potential tool for modulating cellular processes dependent on ROS and redox signaling. Recent evidence from in vitro studies on the effects of NIR in the form of weak (<1 mT) static magnetic fields lends support to this model, where variable field strengths caused alterations in the levels of several species of ROS. Different field strengths resulted in enhanced or suppressed growth of fibrosarcoma cells, which are an in vitro model of connective tissue fibroblast tumors [12]. Additionally, in vivo studies in models of adult tissue regeneration have shown similar effects, where exposure to weak magnetic fields of differing strengths modulated regenerative tissue growth outcomes via changes in the generation of ROS at the wound site after injury [13]. Some field strengths were shown to reduce ROS formation and the subsequent proliferation of adult stem cells, while other strengths promoted both ROS and growth [13]. Although researchers have suggested possible mechanisms by which growth is altered via signaling downstream of ROS, molecular and biochemical clarification is still needed.

The biological effects of NIR on redox signaling and oxidative stress are not limited to cell proliferation, as recent reports suggest NIR may influence apoptosis through a similar mechanism [14]. NIR and its effects on apoptosis are largely associated with, and studied in the context of, adverse biological effects such as cancer [15]. However, regulated cell death is a highly conserved process that is also crucial for proper embryo development, adult tissue homeostasis, wound healing, tissue repair, and prevention of many pathologies [16,17]. In fact, there is a fine balance in the amount of apoptosis that should occur in different tissues at all developmental stages. For example, both excess or insufficient apoptosis has been shown to result in common and debilitating diseases such as Alzheimer’s, Parkinson’s, rheumatoid arthritis, viral infection (such as coronaviruses, HIV, herpes), and carcinogenesis [18,19,20,21,22,23]. Apoptosis can be initiated both intrinsically and extrinsically by a variety of signals, where intrinsic apoptosis involves mitochondria and mitochondrial machinery [24] and extrinsic apoptosis is mediated by activation of transmembrane death receptors (which are part of the tumor necrosis factor (TNF) family) [25,26]. Both the intrinsic and extrinsic pathways are known to be activated by ROS and oxidative stress [27,28], suggesting that tools used to control ROS levels could be used to control levels of apoptosis in vivo through either of these mechanisms.

## 3. Apoptosis and Control of Tissue Growth

Apoptosis has been shown to be a required component of the regenerative response in models such as fruit flies (*Drosophila*), frogs (*Xenopus*), hydra, planarians, zebrafish, salamanders, and the mammalian liver [29]. Despite being a common regenerative process, our understanding of the role of apoptosis as a positive driver of tissue growth remains poorly understood. A counter-intuitive function of apoptosis or programmed cell death is its role in stimulating cell division in neighboring cells through a process known as apoptosis-induced proliferation. Apoptosis-induced proliferation is most commonly discussed in the context of tissue development and tissue repair [30]; however, its potential role in cancer progression and tumor re-emergence following therapy has also been of interest [31]. 

During apoptosis-induced proliferation, apoptosis mediated by caspases causes the release of mitogens (proteins that activate cell division) from dying cells, which then stimulate the proliferation of neighboring cells [32]. Caspases are a family of conserved cysteine proteases that are subdivided into either initiator (Caspase-2, -8, -9, and -10) or effector caspases (Capsase-3, -6, and -7) [33]. Effector caspases, which are activated by initiator caspases, function to carry out the death response. In some contexts, activated effector caspases may also initiate the production of mitogens in dying cells prior to their release [34]. Mitogens that have been reported being released by dying cells include Wingless/Wnt3, Phospholipase A2/Prostaglandin E2, the bone morphogenetic protein (BMP) homolog Decapentaplegic (Dpp), and the epidermal growth factor (EGF) homolog Spitz [34,35,36,37,38,39]. Released mitogens then induce the proliferation of nearby cells through activation of various pro-mitotic signaling cascades, ultimately aiding in the replacement of damaged tissues.

Several mechanisms through which apoptosis-induced proliferation occurs have been identified in a variety of model systems [32], with dependency on ROS arising as a common theme [40,41]. One outstanding question is how the activation of normal cell death (that does not induce tissue growth) and apoptosis-induced proliferation differ. Recent reports highlight the possibility that ROS may be critical regulators of this process [40,41]. ROS can modulate components of redox signaling pathways (such as the mitogen-activated protein kinase (MAPK) pathway, and the phosphatase and tensin homolog (PTEN) and mammalian target of rapamycin (mTOR) pathways), resulting in cell proliferation and growth [42]. While low levels of ROS are associated with the direct induction of cell division and redox signaling, higher levels of ROS are associated with the induction of apoptosis [43]. ROS can initiate apoptosis directly by the activation of caspases through proteolysis at internal sites or redox activation of cysteine catalytic sites [43,44]. ROS can also indirectly induce cell death by controlling the signaling associated with pro-apoptotic pathways. For example, high levels of oxidative stress can cause p53 (a known controller of cell death) to downregulate genes associated with survival, such as Bcl-2 and inhibitor of apoptosis proteins (IAPs). In addition, p53 activates transcription of genes associated with apoptotic induction, like Apaf-1, Fas, and death receptor family proteins [45]. In some cases, ROS-activated p53 has been shown to inhibit the activity of pro-survival proteins or activate translated pro-apoptotic genes, leading to multiple mechanisms by which apoptosis can be regulated [46].

Another well-known target of ROS signaling is the MAPK Jun N-terminal kinase (JNK), which has been strongly associated with the activation of both apoptosis and cell proliferation during wound healing, tissue regeneration, and cancer [47,48,49,50,51,52,53]. Several studies have suggested the effects of JNK expression are context dependent, where transient activation of JNK causes cell proliferation, but sustained activation results in apoptosis [48,54]. In fact, ROS are emerging as a potential regulator of feedback loops that mediate JNK activation, which help make the key decision to die or divide [52,55,56,57]. For example, ROS can activate JNK signaling through the apoptosis signal regulating kinase 1 (ASK1), either by oxidizing regulatory elements like Trx or by activating/deactivating other regulatory proteins [58,59]. JNK activity can also be modulated by ROS oxidation of catalytic cysteine sites on phosphatases that inhibit JNK, such as MAPK phosphatases [60]. Intracellular ROS levels can be increased by JNK [61], which in turn can induce further activity or activate other ROS-related signaling pathways.

Due to their prominent role in controlling apoptosis and related signaling, ROS have emerged as a target for those seeking to understand the mechanisms behind apoptosis-induced proliferation [40,41]. Recent studies of *Drosophila* eye and wing imaginal disc regeneration have found that caspase activity in dying cells leads to ROS generation and subsequent activation of inflammatory cells that initiate a JNK signaling cascade, resulting in apoptosis-induced proliferation [37,38,62]. Similar links between ROS, apoptosis, JNK signaling, and apoptosis-induced proliferation have been seen in other models of tissue regeneration, such as zebrafish fin. ROS generation in the fin within the first 24 h post amputation causes both apoptosis and activation of JNK signaling, which in turn influences downstream Wnt, stromal cell-derived factor-1 (SDF1), insulin-like growth factor (IGF) signaling, and subsequent cell proliferation required for regeneration [51].

Similarly, ROS are also produced by nicotinamide adenine dinucleotide phosphate (NADPH) oxidase following injury in *Xenopus* tadpole tails [63]. Inhibition of either ROS or caspase activity leads to impaired tail regrowth, suggesting that ROS and apoptosis are required for stem cell proliferation during the regenerative process [63,64]. In another study, apoptosis resulting in ROS production in regenerating *Drosophila* wing discs lead to JNK/p38 activation, causing expression of interleukin-6 (IL-6) (part of the Janus kinase (JAK)/signal transducer and activator of transcription (STAT) pathway) and subsequent tissue regrowth [65]. Similar mechanisms of apoptosis-induced proliferation have been implicated in mammalian liver regeneration. There, hepatocytes undergoing apoptosis have been shown to generate ROS, and the produced ROS then facilitate the expression of interleukin-11 (IL-11), causing JAK/STAT activation followed by tissue growth [66].

Taken together, these studies highlight the potential to use ROS manipulation to control new tissue growth through apoptosis-induced proliferation. Because apoptosis-induced proliferation is mediated by redox signaling in many contexts, the possibility exists that NIR may be a novel, non-invasive tool to control apoptosis-induced proliferation in vivo. It should be noted that the existing literature does not distinguish between direct NIR effects on ROS and proliferation (causing cell division in exposed cells) and NIR effects on proliferation via ROS-mediated apoptosis. However, investigating this possible effect of NIR will be crucial both for researchers that are pioneering new regenerative therapies and those pursuing NIR as a potential treatment for cancer, as apoptosis-induced proliferation of cancer stem cells remains a potential off-target effect of its use.

## 4. Potentials of Non-Ionizing Radiation to Control Tissue Growth

As researchers progress towards developing and implementing translational therapies, a need to develop new methods to control proliferation and growth has become increasingly apparent. This growing need has helped spark the global effort to elucidate how tools such as NIR affect biological systems. There is a history of NIR use in the clinical setting both diagnostically (as in ultrasound and magnetic resonance imaging (MRI)) as well as therapeutically (as in transcranial magnetic stimulation (TMS) for depression, diathermy (deep-heating) for physical therapy, and phototherapy for skin disorders) [67]. Therapeutic exposure to NIR has been suggested as an improved and innovative non-invasive approach to influence cellular activities such as growth, proliferation, and differentiation in tissue growth contexts [68]. Yet the mechanisms by which these effects occur are not well understood. Here, we summarize the current literature surrounding how NIR affects ROS, apoptosis, and proliferation, and thus how NIR exposure may be a potential tool for controlling apoptosis-induced proliferation.

An examination of the literature reveals that many different forms of NIR can alter apoptosis. A study of senescence in mice found that treatment with near-infrared light restored organ function by inducing apoptosis of the senescent cells through activation of effector Caspase-3 and -7 [69]. While ultraviolet-C light (UVC, 100–280 nm) is ionizing, both UVA (315–400 nm) and UVB (280–315 nm) are non-ionizing; and studies suggest these non-ionizing forms of ultraviolet (UV) light can affect cellular activities. UVA light therapy was found to enhance caspase-dependent apoptosis when induced by treatment with phytochemicals, effects thought to be due to changes in cellular redox states [70]. Similarly, UVC light therapy in combination with riboflavin was shown to promote ROS generation and increased intracellular calcium concentrations, leading to increased rates of apoptosis in lymphocytes [71]. A study on neuroblastoma (immature nerve cell tumor) cell lines found that exposure to a combination of a 1 mT static magnetic field and a 50 Hz extremely-low radiofrequency field affected proliferation but not apoptosis [72]. A more recent report showed instead that when neuroblastoma and nephroblastoma (kidney tumor) cells were exposed to a combination of a 5.1 mT static magnetic field and a 50 Hz radiofrequency field, there was a decrease in cell proliferation and an increase in apoptosis [73]. To complicate matters, the effects from exposure to weak magnetic fields (<1 mT) may depend on tissue type. When renal cells were exposed to a 500 µT weak magnetic field there was a decrease in apoptosis and proliferation, while astrocytes exposed to the same field strength yielded an increase in apoptotic and proliferative cells [74].

A growing amount of research indicates radiofrequency and static magnetic fields alter cellular activities through changes in ROS levels. Many studies have shown that different tissues exposed to various types and amounts of NIR are differentially affected depending on the context. For example, exposure during mouse embryonic development to a 10 mT field at 50 Hz caused developmental abnormalities by increasing ROS and apoptosis [75]. Similarly, exposure of cardiomyocytes to a 1 mT static magnetic field resulted in increased ROS and induced differentiation through calcium signaling [76]. Although these examples show that static and alternating magnetic fields can increase levels of ROS, other studies indicate that ROS can also be reduced by field exposure. For instance, when a human renal cell line was exposed to a 1 mT, 10 Hz field, production of intracellular ROS was inhibited [77], while exposure of microglial cells to a 1 mT, 50 Hz field prevented elevation of ROS and calcium levels, as well as subsequent cell death [78]. Some studies have even found that NIR exposure did not elicit changes in ROS levels, such as in human amniotic epithelial cells exposed to a 50 Hz magnetic fields ranging from 0–4 mT [79]. For more comprehensive reviews on the different effects of magnetic fields on biological systems and radical pairs, see [80,81].

**Figure 1:**
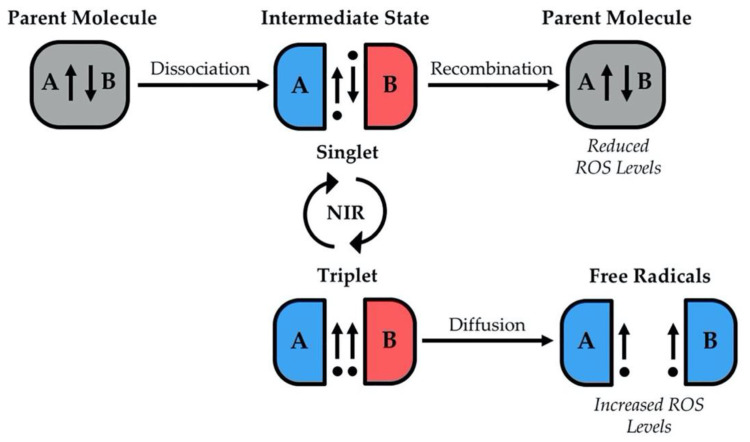
Theoretical model depicting field effects on electron spin state and radical pair recombination. Singlet state is an antiparallel spin and allows for recombination, yielding fewer reactive oxygen species (ROS). Triplet state is a parallel spin and allows for diffusion of radicals yielding more ROS. NIR: non-ionizing radiation.

The literature reflects seemingly conflicting results regarding NIR and biological systems. On the surface, the data demonstrating the effects of NIR exposure on ROS levels and cellular outcomes may seem stochastic. Yet, the reinvigorated field of quantum biology provides possible explanations for these apparent contradictions [82]. Recently, work in theoretical physics has begun to build a functional framework for the mechanisms involved. The predominate theory for understanding how NIR can influence ROS levels revolves around the Zeeman effect and the radical pair mechanism, which are explained in detail in [11,83,84,85,86]. In summary, these suggest that weak magnetic fields can predictably change radical pair recombination rates by altering electron spins through changes in angular momentum (known as spin state theory). This model suggests that weak magnetic fields may alter ROS levels by promoting either recombination or diffusion of free radicals (Figure 1). When a parent molecule dissociates into radical pairs, whether they will recombine or not lies in accordance with the spin states of the valence electrons. For recombination to occur, the unpaired electrons on radical pairs must have opposing (antiparallel) valence spins, known as the singlet state. When the valence spins are parallel (triplet state), recombination cannot occur. Importantly, when NIR interacts with radicals, it can change their spin state. For ROS, this suggests that some field strengths should promote the singlet state/recombination yielding fewer reactive species, while other field strengths should promote the triplet state/dissociation yielding more reactive species.

Two fibrosarcoma cell culture studies demonstrate this phenomenon. When a fibrosarcoma cell line was exposed to a combination of a 45 µT static magnetic field and a 10 MHz, 10 µT weak radiofrequency magnetic field, there was a significant increase in the production of ROS but a reduction in cell proliferation [12,87]. However, when fibrosarcoma cells were exposed to weak static magnetic fields across a range from 0–600 µT, ROS levels and proliferation were either increased or decreased depending on field strength, as predicted by theoretical modeling [12,87]. These binary/opposing effects have also been found in vivo. Our own work indicates that exposure to weak magnetic fields from 0–600 µT is able to either inhibit or promote ROS levels in regenerating planarians, modulating cell proliferation, differentiation, and tissue growth after injury in a field-strength dependent manner [13]. Therefore, consistent with the spin state model, certain NIR exposures can and do lead to opposite effects in the same tissues as compared to other exposures.

In summary, the extant data demonstrate that NIR is able to produce biologically significant changes in ROS levels, apoptosis, and/or cell proliferation. Furthermore, there are well-established links between ROS and apoptosis, ROS and proliferation, and apoptosis and proliferation in the context of development and tissue growth. These links suggest that modulation of ROS levels is one mechanism by which NIR elicits these cellular changes. Because of the many connections between NIR, ROS, apoptosis, and proliferation (as summarized in Table 2), we propose that NIR exposure might function specifically through apoptosis-induced proliferation.

## 5. Conclusions

**Figure 2:**
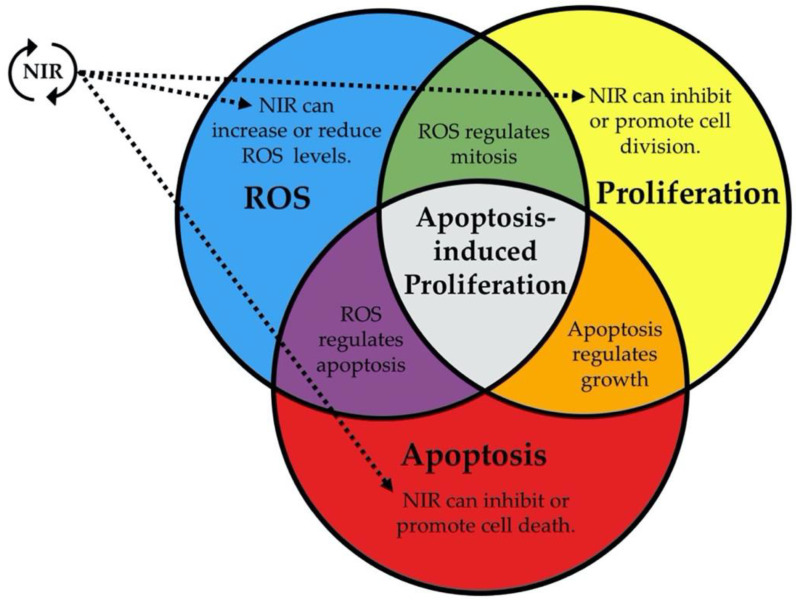
Relationships between key concepts. Overlap between reactive oxygen species (ROS) signaling, apoptosis, and cell proliferation (Venn diagram), overlaid with the known effects from non-ionizing radiation (NIR) exposure (dotted lines).

**Figure 3:**
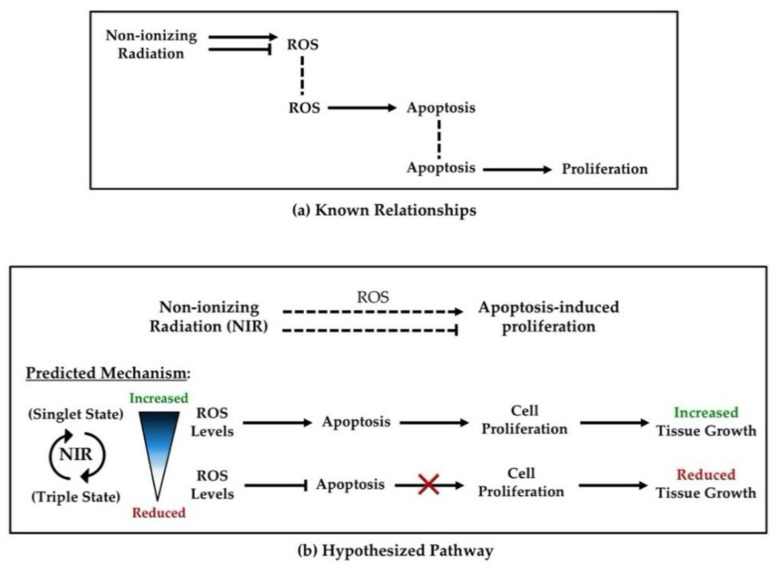
Diagram of known and proposed interactions. **(a**) Known relationships from the literature between non-ionizing radiation (NIR) and reactive oxygen species (ROS); ROS and apoptosis; apoptosis and cell proliferation. (**b**) Hypothesized pathway by which non-ionizing radiation could modulate apoptosis-induced proliferation via changes in ROS levels. Barred lines: inhibition. Arrows: activation. Dotted lines: predicted interactions.

Recent research on NIR has shown that it can be an effective, non-invasive approach to control proliferation and tissue growth; however, few studies have robustly investigated the mechanisms by which these effects occur [88]. This review has highlighted the evidence present in the literature that demonstrates NIR may be useful as a tool to manipulate apoptosis-induced proliferation (Figure 2). The evidence suggests that NIR could modulate apoptosis-induced proliferation and, thus, tissue growth due to the following: (1) NIR can influence levels of ROS through alteration of electron spin-states, (2) ROS levels can regulate apoptosis through redox signaling, and (3) apoptosis can drive new tissue growth by causing cell proliferation in surrounding cells via activation of mitogenic pathways (Figure 3). While the possible applications are exciting, we currently lack studies that connect these known relationships from the literature, highlighting the need for future investigations. Research exploring these potential effects will be required before NIR can be seriously considered as a tool to promote new tissue growth for regenerative medicine or inhibit growth for cancer therapeutics, as uncontrolled apoptosis-induced proliferation could potentially result in carcinogenesis [32,40]. Given the immense potential benefit and the innovative treatments that may result in the future, we argue that investigating the effects of NIR on apoptosis-induced proliferation is a line of research that must be pursued.

## Figures and Tables

**Table 1:** Brief definitions for some of the terminology used in this review.

Terminology	Definition	Units (Symbol)
Reactive Oxygen Species (ROS)	A chemically reactive class of molecules containing oxygen.	-
Free Radicals	Highly reactive atoms or molecules characterized by a lone (unpaired) valence electron.	-
Spin State	A description of valence electron position, important for determining recombination or disassociation of radical pairs.	-
Non-ionizing Radiation (NIR)	Radiation that contains too little energy per photon to ionize an atom or molecule (remove an electron). Wavelengths greater than 280 nm.	Nanometers (nm)
Zeeman Effect	The splitting of spectral lines (electron decoupling) due to a static magnetic field.	-
Electromagnetic Fields (EMFs)	Radiation produced by the movement of charge. The interaction of electric and magnetic fields is generally described as a wave form transporting energy.	Varies
Magnetic Fields (MFs)	A vector field emanating from magnetic material or the result of electrical current.	Tesla (T) & Hertz (Hz)
Static Magnetic Fields	A magnetic field that has a constant or unchanged vector.	Tesla (T)
Radiofrequency	Refers to the osculation of electrical current or EMF. Magnetic fields with radiofrequency are produced by alternating electrical current.	Hertz (Hz)
Apoptosis-induced Proliferation	Cell division by mitosis (proliferation), induced by mitogen release from neighboring cells undergoing programed cell death (apoptosis).	-

**Table 2:** List of non-ionizing radiation (NIR) studies and effects on reactive oxygen species (ROS) and cellular processes. This list includes only those studies referenced in this review and is not meant to be comprehensive. WMFs: weak magnetic fields. MFs: magnetic fields. RF: radiofrequency. EMF: electromagnetic fields. ES: embryonic stem (cells).

Model System		NIR Type	Effects on ROS	Effects on Cellular Activities
Fibrosarcoma Cells, HT-1080 Line		Static MFs [12]	Varied based on strength	Varied proliferation effects
		Weak RF MFs [87]	Increased ROS	Decreased proliferation
Epithelial Cells	HaCaT, A431, and A549 Cell Lines [70]	Ultraviolet light	Increased ROS	Decreased proliferation, increased apoptosis
	A549 Cell Line [14]	Static MFs	Increased ROS	Decreased proliferation
	FL Cell Line [79]	Static & RF WMFs	No effects	No effects
Mouse ES Cells	CCE Cell Line [75]	Static & RF MFs and WMFs	Increased ROS	Increased apoptosis
	CGR8 Cell Line [76]	Static MFs		Decreased apoptosis
Neuroblastoma Cells	Lan-5 Line [72]	Static MFs	*-*	Increased proliferation, no change in apoptosis
	CHLA-255 & N2a Lines [73]	Static & RF WMFs		Decreased proliferation, induced apoptosis
Nephroblastoma Cells, G401 Line		Static & RF WMFs [73]	-	Decreased proliferation, induced apoptosis
		Static MFs [14]	Increased ROS	Decreased proliferation
Renal Tubular Epithelial Cells, HK-2 Cell Line		EMFs [77]	Decreased ROS	Decreased apoptosis
Leukemia Cells, THP-1 Cell Line		Static MFs [3]	Increased ROS	Decreased proliferation, increased apoptosis
Lymphocytes, (whole blood, ABO/D matched)		Ultraviolet light [71]	Increased ROS	Increased apoptosis
Microglial Cells, HM06 Line		EMFs [78]	Decreased ROS	Decreased apoptosis
Nephroblastoma G401 Cells in Mice (in vivo)		Static & RF WMFs [73]	*-*	Decreased tumor mass
Regenerating Adult Planarians (in vivo)		Static WMFs [13]	Decreased ROS	Decreased proliferation, inhibited regeneration

## Data Availability

Not applicable.
